# Migrant-Led Community Organisations: Mobilising Ethnic Capital to Support Refugees and Asylum Seekers in England

**DOI:** 10.3390/bs16010136

**Published:** 2026-01-17

**Authors:** Samson Maekele Tsegay, Zewdi Amanuel Dagnew

**Affiliations:** School of Education, Anglia Ruskin University, Cambridge CB1 1PT, UK

**Keywords:** migrant-led organisations, ethnic capital, grassroots humanitarianism, refugees, asylum seekers

## Abstract

Refugees and asylum seekers (RASs) are among the most marginalised, vulnerable, and economically disadvantaged groups worldwide. As a result, many government and non-government organizations, including migrant-led community organizations (MLCOs), support RASs to improve their lives in their host countries. However, there is a lack of research on the role and challenges of MLCOs supporting RASs. Therefore, informed by the concepts of grassroots humanitarianism and ethnic capital, and based on data collected through focus group discussions, this article explores the rationale, activities, and challenges of Eritrean MLCOs in England. The findings indicate that MLCOs help fill some gaps left by government agencies by providing RASs with strong advocacy and support systems to protect their rights and meet their needs. Although volunteers with limited funding run these organizations, they utilise ethnic capital to provide knowledge, raise awareness, and deliver culturally sensitive services to RASs in their own language. This article advances understanding of MLCOs’ work and improves their services to better meet the needs of RASs. It also contributes to knowledge by highlighting MLCOs’ role as sites of learning and education.

## 1. Introduction

Migrant-led community organisations (MLCOs) are nonprofit groups, often grassroots, run by and for refugees, asylum seekers, and other migrants. They are a vital part of the UK’s third sector, filling service gaps left by government agencies, especially in the hostile environment (Turcatti et al., 2024). Despite limited funding and staffing (Turcatti et al., 2024), MLCOs play essential roles in creating caring spaces for refugee and other migrant families by engaging in civic activism to influence the migration policies of receiving countries (Sahin Mencutek, 2021) and serving as a bridge between immigrants and the settled population (Martinez-Damia et al., 2024). Many MLCOs offer services aimed at improving the socioeconomic and cultural experiences, as well as supporting the mental well-being of RASs (Tsegay, 2020; Turcatti et al., 2024). This is significant given the large number of RASs worldwide, including in the UK.

According to UNHCR (2024), there are 43.7 million refugees, 6.9 million asylum seekers, and 4.4 million stateless people worldwide. This includes 365,300 refugees and 99,790 asylum seekers hosted in the UK (Home Office, 2024). Asylum seekers are individuals who seek international protection in other countries but have not yet received legal recognition or status (Tsegay, 2023). They are called ‘refugees’ when their asylum application is accepted and, hence, receive international protection due to a threat of persecution because of their race, religion, nationality, membership of a particular social group, or political opinion, and lack the protection of their own country (Tsegay, 2023; UNHCR, 2011). The UK is one of the top destination countries in Europe for Eritrean migrants. Although there is no exact census, there are more than 40,000 Eritrean refugees and British Eritreans in the UK (Tsegay, 2020).

Refugees and asylum seekers (RASs) flee from war, forced military conscription, and religious and political persecution (Burnett & Peel, 2001; Tsegay, 2023). Therefore, they are often forced to leave their countries, undertaking long, risky journeys to seek asylum in the Global North, including the UK (Tsegay, 2022). However, most of their asylum applications are delayed, and the loneliness they experience increases, making them vulnerable to anxiety and psychological distress (Tsegay, 2022). Additionally, many RASs face harassment, hate speech, racism, and uncertainty due to changes in immigration laws (Boukari & Devakumar, 2024; Hargreaves, 2016; Kalt et al., 2013; Thompson et al., 2018). The rising number of RASs in the UK has led to a series of anti-immigrant policies and protests, which Boukari and Devakumar (2024) describe as demonising and dehumanising. These racist and xenophobic policies and practices not only create fear among migrants but also bring back traumatic memories and impact their socioeconomic and cultural integration (Fernandez-Reino & Cuibus, 2024).

Furthermore, the socio-cultural and economic integration of RASs has become a concern for the local population, as many argue that a significant number of refugees are not integrating into the UK, mainly due to language barriers (Lapshynov, 2025; UK Parliament, 2024). However, it is important to recognise that RASs face complex challenges (Bhatia & Wallace, 2007; Ruokolainen, 2022; Sandri, 2018; Thompson et al., 2018; Tsegay, 2023) due to the hostile conditions in host countries and the traumatic experiences they endure before migration and during dangerous journeys to reach them (Burnett & Peel, 2001; Hargreaves, 2016; Kalt et al., 2013; Thompson et al., 2018). These factors lead to increased psychological stress and heightened social needs (Carlsson et al., 2014; Mahon, 2022; Thompson et al., 2018). Furthermore, accessing healthcare, employment, and other essential services is often influenced by citizenship status, language, and cultural barriers, which worsen their vulnerability and further stigmatise their social standing (Bhatia & Wallace, 2007; Bloch & Schuster, 2002). Additionally, the uncertainty related to asylum procedures, along with economic hardship and social exclusion, puts asylum seekers in a fragile situation, impacting their mental health (Auyero, 2020; Busetta et al., 2021; Hynie, 2018; Tsegay, 2022). These circumstances often lead RASs to depend on non-governmental organisations (NGOs) and civil society for support, as government efforts mainly focus on basic needs, especially food, housing, and medical care (Hollifield, 2004; Tsegay, 2020).

As shown above, RASs are among the most marginalised, vulnerable, and economically disadvantaged populations worldwide, including in economically developed Western countries (Langmead, 2016; Tsegay, 2020). Therefore, many national and international organisations, such as the UNHCR, have been established to support RASs and improve their lives by providing emergency services, legal protection, policy advocacy, and increased knowledge and awareness (Adebayo et al., 2024). Additionally, several MLCOs offer RAS services in a culturally sensitive manner, often using their native language. However, most of these organisations have limited interaction with each other, and many refugees are unaware of their services. This article aims to examine the rationale and activities of Eritrean MLCOs in England and explore the roles and challenges they encounter in implementing them. The following three research questions guide this article:

1. What is the rationale for establishing Eritrean MLCOs in the UK?
2. How do the Eritrean MLCOs support refugees and asylum seekers in England?
3. What are the challenges that Eritrean MLCOs face in England?

The article draws on the concepts of “grassroots humanitarianism” (Dolska, 2022; Sandri, 2018) and ethnic capital (Borjas, 1992; Kim, 2019), as well as data collected through focus group discussions with leaders of Eritrean MLCOs in England. The article is significant because it contributes to improving the socio-cultural and economic experiences of RASs through MLCOs. Moreover, the article helps to better understand the MLCOs’ work and improve their services to meet the needs of RASs.

## 2. Grassroots Humanitarianism

This article uses the concept of grassroots humanitarianism to understand the nature, work, and challenges of MLCOs. Grassroots humanitarianism, also known as grassroots volunteerism, refers to efforts initiated by informal and small-scale organisations that emerged to address the worsening conditions of RASs (Dolska, 2022; Sandri, 2018). These grassroots organisations, mostly apolitical, funded by public donations and operated by volunteers, are driven by the urgent need to provide essential aid and services, such as distributing donations, building shelters, and organising social activities (Muehlebach, 2019; Sandri, 2018). For example, Play4Calais focused on providing pop-up cinemas, safe play areas, sports, and essential aid to refugees, especially women and children, offering brief moments of escapism from their trauma (McGee & Pelham, 2018). Similarly, the Refugee Youth Service created safe spaces for children and expanded its mission to include child safeguarding, rights advocacy, education, and legal and psychological support. Both groups operated informally, guided by a moral commitment to human rights and a rights-framed approach rather than a formal institutional framework (Hilhorst & Jansen, 2012; McGee & Pelham, 2018; Miller, 2010). This indicates that grassroots organisations and their staff see themselves as humanitarian workers, guided by human rights and humanitarian principles in both their language and actions, such as providing humanitarian aid (Hilhorst & Jansen, 2012). They consider non-discrimination a core part of their work with disadvantaged groups and individuals, like RASs (Miller, 2010).

With the emergence of hostile immigration policies, many grassroots organisations also began campaigning for “change to asylum policies and for more humane treatment of refugees” (Sandri, 2018, p. 66). In Brussels, the Citizens’ Platform for the Support of Refugees faced a temporal dilemma in their efforts to support around 500 stranded migrants in 2017 (Vandevoordt & Fleischmann, 2021). While they provided immediate humanitarian aid, such as shelter and legal information, they also advocated for structural solutions, including the establishment of a reception center and the use of the sovereignty clause to circumvent European Union regulations. Many grassroots organisations, such as “No Name Kitchen,” also collect testimonials to document the border and immigration abuses faced by RASs (Augustova & Sapoch, 2020). This tension between addressing urgent needs and pursuing long-term political change shows how grassroots organisations navigate complementary timelines, balancing immediate actions with future reforms, and creating opportunities for significant social and political change (Lafaut & Coene, 2019).

As noted earlier, many grassroots organisations offer a variety of services that link humanitarian efforts with activism. They provide humanitarian aid, social and political activism, and integration support for RASs, creating new social spaces marked by compassion, empathy, and solidarity (Muehlebach, 2019; McGee & Pelham, 2018; Vandevoordt, 2019). However, such activities put grassroots organisations in conflict with the violent borders of states (McGee & Pelham, 2018) and their proponents, such as anti-immigrant media, extremists, and political organisations (Butler, 2025). Butler (2025) reported that many voluntary organisations in the UK have started implementing increased security measures to protect staff, beneficiaries, and property due to rising intimidation and threats of violence regarding their support for immigrants. This shows that the abuse and fear experienced by RASs have intensified to affect grassroots volunteers, including locals, who show solidarity with one of the world’s most vulnerable communities.

Overall, despite their limitations and challenges, grassroots organisations effectively mobilise volunteers and resources, providing essential services and support to RASs. In doing so, they demonstrate the powerful impact of community-driven efforts during crises and become a beacon of hope for RASs in times of need. Additionally, these grassroots initiatives reflect a commitment to solidarity and human dignity, demonstrating that meaningful change can originate from the ground up, even in the most difficult circumstances. However, the adoption of human rights frameworks places them at odds with the violent border regime and practices.

## 3. Ethnic Capital

As indicated above, MLCOs are part of grassroots humanitarianism, mostly operated by migrants for the benefit of RASs and other migrants. Hence, they mostly mobilise ethnic capital to provide culturally sensitive support (Rigazio-DiGilio & Ivey, 2007). Ethnic capital is the “collective human and/or social capital produced and utilized by ethnic group members”, which determines their prospects for socioeconomic development (Kim, 2019, p. 359). Despite the use of ethnic capital in migration studies, scholars have mainly focused on its role in students’ educational achievement from migrant backgrounds (e.g., Iqbal & Modood, 2023; Modood, 2011, 2012; Postepska, 2019; Shah et al., 2010). Less attention has been given to the explanatory power of ethnic capital for migrants’ socio-cultural and economic development, as well as mental well-being. The limited studies available mainly explore the relationship between ethnic capital and intergenerational mobility (Borjas, 1992; Postepska, 2019). This article uses the concept of ethnic capital to examine the rationale, role, and challenges of MLCOs.

Pierre Bourdieu’s work is commonly used to analyse how capital—social, economic, cultural, or symbolic—(Bourdieu, 1986) influences migrants’ access to economic resources, enhances their cultural awareness, and increases their sense of belonging with the host society and its institutions (see Tsegay, 2020). However, as Shah et al. (2010, p. 1111) noted, “ethnicity is not just part of the cultural and symbolic schema of groups but has material impact.” Ethnic institutions and social relations can act as channels for cultural and social capital (Shah et al., 2010). This indicates that migrant-led community organisations (MLCOs) support and encourage RASs in gaining the social and cultural capital necessary to improve their experiences in their host countries.

Ethnic capital consists of elements of human, social, and cultural capital that enhance the skills and experiences of a specific ethnic group (Kim, 2019; Shah et al., 2010). For instance, ethnic cultural capital involves cultural practices that foster favourable conditions for economic growth and progress (Levanon, 2011). This is vital for RASs, given their vulnerable status in their host countries. The delays in asylum decisions and the dispersal program for RASs, regardless of their geographical preferences, isolate them from their social networks, leading to loneliness and depression (Darling, 2022; Tsegay, 2020). Such exclusionary measures also impact the social capital of RASs because “the volume of the social capital possessed by a given agent partly depends on the size of the network of connections he can effectively mobilise” (Bourdieu, 1986, p. 249). Therefore, MLCOs are expected to develop social networks that can serve as a foundational means of support for RASs, ensuring better socioeconomic development and mental health.

Ethnic capital influences social networks, which refer to how individuals, organisations, or groups interact with others within their networks (Borgatti & Halgin, 2011). For example, Levanon (2011) argued that ethnic social capital impacts immigrants’ economic activities by providing information, training, and credit they might not otherwise have access to. A migration network is a collection of interpersonal relationships in which migrants, including RASs, interact with one another (Haug, 2008). However, MLCOs’ social networks go beyond internal migrant relationships, as they also connect with people, organisations, or groups from other ethnic backgrounds, including local communities. In this context, ethnic capital considers MLCOs as providers of vital resources that can support fellow RASs through material or social means (Kim, 2019; Shah et al., 2010). It is also important to recognise that migration experiences, along with social, cultural, and linguistic similarities, enable effective and meaningful support (Rigazio-DiGilio & Ivey, 2007).

## 4. Methodology

This article is based on a qualitative study. The study used focus group discussions for data collection and thematic analysis for data analysis. It also followed relevant ethical guidelines throughout the research process.

### 4.1. Data Collection and Analysis

**Focus Group Discussion:** We conducted five focus group discussions with leaders or active members of Eritrean MLCOs in England. Focus group discussion was employed to investigate and gain deep insight into the roles and challenges of MLCOs in supporting asylum seekers and refugees in England (Bryman, 2016). Purposive sampling was employed to select participants based on their roles and experiences, addressing the case under study (Stratton, 2024). Accordingly, leaders of Eritrean MLCOs in England were approached to participate in focus group discussions, which lasted approximately an hour. Although we planned four focus groups with 4 to 6 participants each, we ended up with five focus groups from nine MLCOs, each with 2 to 4 participants, because it was challenging to find a single time slot that worked for everyone. In total, 14 participants from 9 Eritrean MLCOs participated in the focus groups (see Table 1 below).

The focus group discussion was conducted online via Microsoft Teams or Zoom in Tigrinya (Eritrean language) to facilitate better communication and exchange of ideas. Participants were asked about the objectives and activities of the MLCOs, as well as the challenges they face. The researchers are fluent in Tigrinya and English, which facilitated data collection, transcription, and translation into English. The focus group was audio-recorded with participants’ permission, and the audio was transcribed and translated into English by the researchers to ensure rigorous data analysis.

**Data Analysis:** Thematic data analysis was employed to analyse data collected through focus group discussions, as it summarises key features and provides a detailed interpretation of the data (Bryman, 2016; Braun & Clarke, 2006). The approach is a valuable tool for focus group data because it helps researchers identify, analyse, and report themes within the data, offering meaningful interpretations of different perspectives shared by group participants (Hecker & Kalpokas, 2024). Specifically, we followed Braun and Clarke’s (2006) six phases for conducting thematic analysis: familiarising with the data, generating initial codes, searching for themes, reviewing themes, defining and naming themes, and writing up. The research questions were used to generate codes and organize them into themes, while the theoretical frameworks supported the data analysis, enabling the drawing of realistic conclusions.

### 4.2. Ethical Considerations

The research project adhered to Anglia Ruskin University’s ethical guidelines and obtained ethical approval from the School of Education and Social Care Research Ethics Panel (ETH2324-0316). Specific attention was dedicated to issues of confidentiality, anonymity, and informed consent throughout the research process. Participants were provided with a consent form that included an information sheet about the project and were given the opportunity to ask questions before giving their consent. A translation copy of the participant information sheet and consent form was also prepared in Tigrinya to help participants with English-language reading and comprehension. The digital recordings and transcripts were kept in a secure, password-protected area, accessible only to the research team. Moreover, the transcripts were anonymised, and any details that could identify the participants were removed. Confidentiality and anonymity were maintained by using pseudonyms when reporting the findings, including the writing of this article.

## 5. Findings

### 5.1. Rationale for Establishing MLCOs

UK migration policies are underpinned by the expectation that refugees and asylum seekers (RASs) should integrate into the country (Tsegay, 2020, 2022). However, like many other host countries, the UK does not provide adequate and relevant services that help RASs understand their new environment, access essential services, and become productive members of the established society (Mayblin & James, 2019; Tsegay, 2020). The study participants reported that Eritrean MLCOs offer advice, information, and advocacy services related to immigration, integration, and well-being of RASs. In fact, many of the MLCOs were established to address challenges faced by Eritrean RASs. For example, Basar and Bereket said:

*I worked as an interpreter, and I witnessed many Eritrean migrants, including women with children, suffering social isolation, stress, and depression. I was always thinking of a way to meet the women. The only chance was to meet them at church, where they could gather; however, that was not easy either. Then, in collaboration with the Eritrean community in the city, we established CO2, and I got a chance to meet with the women in the community once a week. We meet at the community centre, gather, have coffee and tea, and socialise while exchanging ideas. We talk about different things, including postpartum depression and mental well-being.*

(Basar)

*The idea to establish CO3 was initiated after we heard about the suicide of about three Eritrean refugees. It was aimed at ways to reduce mental health issues by supporting them in housing issues, job finding, etc. So, we agreed to set up an organisation as a mentorship charity organisation, with all the work done by volunteers.*

(Bereket)

Basar and Bereket were among the founding members of their respective organisations. Both organisations have expanded their activities to include immigration, integration, and advocacy services to empower RASs to receive the attention, information, and support they need to create a home away from home. They partly address gaps created by UK immigration policies, such as delays in asylum decisions and restrictions on work permits.

Expanding on the previous narratives, Sesen explained that the reason for founding CO4 stemmed from her own experiences as an asylum seeker and later refugee in the UK, where she felt loneliness and exclusion. She believed it would be helpful to have a charity organisation that could support Eritrean migrants, especially new RASs, with assistance from fellow migrants who are fluent in English and have a better understanding of UK culture.

*First, the plan was to meet online via Zoom and invite professionals from various fields, including mental health, integration, and postpartum depression, to share their expertise with the group. However, the long-term plan is to have a community centre where people can get support and the right information on immigration, employment, health, housing, and education, particularly for new refugees and asylum seekers. We also signpost those who need further assistance to the relevant organisation.*

(Sesen)

Elen and Sina also echoed Sesen’s idea that she had been observing refugees experiencing mental health issues such as stress and depression due to their dangerous journey to reach the UK. Similarly, Keria explained that CO9 was established to tackle violence against women and girls that she witnessed in the UK. Ellen and Keria elaborated:

*Awet and I had the idea of supporting refugees in mental health and other issues that refugees experience for a long time. And it all started from our experience and the problem we were observing, like many young adults were developing mental illness and becoming drug addicts. There was also a huge problem with new refugee families.*

(Elen)

*We are involved in the community, and we were observing women’s rights being diminished, and women being violated and abused. As most of them are young, have low levels of education, and are in a new country, they do not know their rights. These things led us to form the organisation and support women.*

(Keria)

Sesen, Elen, and Keria believed that RASs were not receiving sufficient government support, especially since many could not speak English and were unfamiliar with UK culture and systems. Therefore, they needed someone to act as a bridge, helping Eritrean RASs access relevant information, advice, and support. In these situations, MLCOs were viewed as a way to recruit volunteers and galvanise the community to work toward a common goal: organising activities to improve the experiences of Eritrean RASs in the country, particularly by addressing gaps left by the UK government. However, as discussed below, the findings reveal that MLCOs cannot address all the challenges of RASs since some issues require policy changes.

### 5.2. MLCOs’ Activities and Support

The findings indicate that Eritrean MLCOs engage in a range of activities that support RASs in developing themselves and improving their experiences in the UK. The activities can be divided into four interrelated categories: community awareness and inclusion, advocacy, guidance and counselling, and skill development.

#### 5.2.1. Community Awareness and Inclusion

Many MLCOs view community awareness and inclusion as their primary task, believing that RASs need to understand their host society and environment to access relevant services and enhance their experiences. Hence, they provide training, advice and information, and organise workshops to promote socioeconomic empowerment and mental well-being, thereby facilitating psychosocial support and inclusion.

*Our organisation supports refugees and asylum seekers with educational, employment (job-seeking), welfare, and housing issues. During COVID, all organisations were working online, which created inaccessibility to support and provide information due to digital illiteracy and a lack of smart devices, particularly among asylum seekers. Since big organisations have some incongruence between their policies and the actual situation of refugees, such as their capabilities and language barriers, we were supporting refugees and asylum seekers in person, following social distancing and other COVID-19 prevention strategies. At that time, we were visiting asylum seekers in different hotels, and they were very distressed, looking like prisoners. We were able to provide them with traditional food and stay some time with them.*

(Teages)

*Most of our services involve providing guidance and information, and our volunteers offer consultation based on their professional backgrounds. We also signpost those who need further assistance to the right organisation. At first, we were giving educational sessions online. Now we have different in-person events like mental health support, immigration support, housing, and education, particularly for asylum seekers in hotels and dispersals. We do this in partnership with different organizations like the NHS and other MLCOs. Our online presence also benefits people everywhere, globally. However, in the UK, people from London, Manchester, Leeds, and Birmingham contact us through Facebook and WhatsApp.*

(Sesen)

Teages’s and Sesen’s narratives indicate that MLCOs provide RASs with relevant information and guidance on mental health, immigration, housing, employment, and education in their own languages. Similarly, Hanna mentioned that CO9 has also provided asylum seekers with traditional food, shoes, clothes, and prepaid phone cards. Asylum seekers receive a limited allowance (£49.18 per person per week) to cover essential living needs, including food and clothing (Gower, 2021); they are not allowed to work for at least the first twelve months (Gower et al., 2024). The MLCOs’ support mechanisms are essential for RASs, especially those who have no family members in the UK and are dispersed in remote areas where there are few or no Eritrean migrants. As Teages noted, many RASs cannot communicate effectively online due to language and digital barriers, including a lack of smartphones and digital literacy, especially among new arrivals. Therefore, the activities and support of MLCOs are vital in bridging the gap between RAS needs and available services. They also provide additional support to fill gaps not covered by government agencies.

Although most Eritrean MLCOs provide comprehensive support for socioeconomic, cultural, and mental well-being, few organizations, such as CO9 and CO6, offer assistance to specific groups based on gender, ethnicity, or religion.

*Our organisation is primarily focused on women and girls. The main aim of our organisation is to empower women, especially asylum seekers and new refugees, raise awareness of their own and their children’s rights, and raise awareness about harmful practices like FGM, forced marriage, and gender-based violence. Our work is not limited to one city; it also extends across the UK. We liaise with organisations that work with women or other specific needs, depending on the clients’ issues.*

(Keria)

*Our organisation aims to empower and mobilize our community in the UK to transform their lives and preserve their cultural heritage. We achieve this by offering community-centred programs and services that address their socioeconomic, intellectual, and cultural needs. We strive to increase their civic participation, protect their integrity as a community, and contribute to broader society in the UK.*

(Habtom)

Keria’s and Habtom’s quotes suggest that Eritrean MLCOs can be established for specific groups, such as a specific gender or ethnic group. This is not intended to alienate them from the larger Eritrean community; instead, as Habtom explained, it is to focus on ‘restorative integration’ of Eritrean RASs in the UK (see Aldegheri et al., 2025). Restorative integration is also crucial for women, who often experience violence both during their journey and in their host countries (Tsegay & Tecleberhan, 2023), because this approach helps restore human dignity that is threatened by migration, including during the migration journey and asylum process (Aldegheri et al., 2025). Although MLCOs can deploy ethnic capital to support the integration of RASs, integration is a multidimensional and multidirectional process that requires participation and adjustment from both RASs and the host society (Commission on the Integration of Refugees, 2024).

Most MLCOs have online platforms and WhatsApp groups, and they livestream some of their information and workshop sessions on Facebook and/or YouTube to benefit those who cannot attend in person. For example, CO3 and CO4 have YouTube channels and invite guest speakers to share their experiences on various issues relevant to Eritrean RASs. Additionally, as Sesen mentioned, MLCOs mainly rely on volunteers who support RASs through their personal and professional experiences. They also direct RASs to other relevant organisations, such as the NHS for mental health issues and immigration solicitors for immigration cases. This indicates that the MLCOs are grassroots organisations, mostly rooted in migrants’ ethnic capital, to enhance RASs’ cultural awareness and improve their socio-cultural and economic integration in the UK.

#### 5.2.2. Advocacy

Advocacy is a complex term with various definitions, the main one being a commitment to work with other institutions to achieve lasting changes in policy and practice (Ross, 2013). In the context of RASs, advocacy involves helping them address immediate safety concerns and basic needs, such as food, housing, and mental and physical health services (Soken-Huberty, 2023). The participants mentioned that the MLCOs not only engaged in immigration and gender-based violence advocacy but also provided direct support to enhance refugees’ and/or asylum seekers’ experiences during crisis.

*CO4 is doing a great job of impacting many refugees’ and asylum seekers’ lives. Particularly in the Rwanda issue, many asylum seekers were emotionally and mentally distressed, and CO4, in collaboration with other MLCOs, supported many by giving information and addressing their emotional conditions. We were by their side, becoming their voice and supporting them in explaining their case and in obtaining relevant support from GP and other service providers. Those who were detained were shocked at first. We tried to call them and also visited those in London.*

(Sesen)

Sesen’s excerpt shows that many RASs face fear, harassment, and mental health issues in their host countries due to changes in immigration policies, racism, and harsh treatment. As mentioned earlier, a clear example is the UK’s Rwanda deportation plan (Parker & Cornell, 2024), as well as UK political discourse and far-right protests, which caused panic among many RASs and renewed their fears (Askew, 2025). Therefore, MLCOs organized Eritrean and other communities to advocate for RASs during times of hardship. They also work with other organisations to coordinate peaceful protests and online petitions in response to unfair RAS treatment and policies.

In particular, during the UK–Rwanda partnership to send asylum seekers, many Eritrean MLCOs, such as CO1, CO2, CO3, CO4, and CO9, formed a partnership, creating collective advocacy to amplify their campaign and advance their support for asylum seekers. The findings show that MLCOs like CO8 (Zemen) and CO9 (Keria) visited detention centres where many Eritreans were held, while CO3 (Bereket) and CO1 (Sina) worked on digitising and recording asylum seekers’ documents and translating their cases from Tigrinya to English. Other members of CO3 (Awet) and CO4 (Sesen) also offered immigration and emotional support, as many asylum seekers had to sign their names as required by the Home Office but were afraid of being detained or deported, which has been part of the UK’s “hostile environment approach to asylum seekers for some time” (Parker & Cornell, 2024, p. 3). Additionally, as Keria mentioned, women and girls are more vulnerable because they experience gender-based violence, especially from fellow RASs, including their partners (Tsegay & Tecleberhan, 2023). Therefore, the MLCOs work not only to prevent violence against women and girls but also to address its root causes through education and campaigns.

*We advocate for women/girls and families in different ways. We accompany them to lawyers (online or in person) and sometimes attend court when necessary. We advocate for refugees and asylum seekers to get relevant services, such as mental health services. We also support single women at schools, especially when their children have special needs.*

(Keria)

Zemen and Finhas argued that Eritrean MLCOs focus on Eritrean RASs, not because they are not interested in other communities, but because of their socio-cultural and experiential familiarity with the Eritrean cases and their limited capacity to reach a wider group of communities. For example, Zemen explained:

*It is not to discriminate, but they want someone from their own country, with the same culture and language, which creates trust, particularly on culturally sensitive issues. It is not only what they say, but also their body language, that reveals their situation. Others were also referring Eritrean cases to me because of their complexity, because of our focus on Eritreans. Our presence makes people with different issues, particularly domestic violence victims, come to our workplaces mentioning our names. And these keep us working and supporting them.*

(Zemen)

Zemen emphasised the importance of ethnic capital, such as social and cultural capital and proximity, in handling cases of refugees and asylum seekers. It greatly influences the services MLCOs provide to Eritrean migrants in their native language. Keria, another participant, noted that women and girls, especially victims of domestic violence, visit MLCOs seeking advice and support, as they are caught between their pain and cultural pressure (or lack of awareness) not to report their partners. Therefore, they seek help from someone who can understand their complex situation and support them in navigating it.

#### 5.2.3. Guidance and Counselling

One of the prominent examples that participants mention regarding the role of MLCOs is guidance and counselling. Sesen and Awet argue that, despite their post-traumatic stress disorder and other mental health issues caused by their dangerous journey and asylum process, Eritrean RASs do not receive adequate guidance and counselling services from British mainstream institutions. However, MLCOs have made a big difference in supporting RASs through peer counselling.

*Traditionally, everyone is an advice provider, but to be a mental health supporter (peer support), you need more training in the principles of peer support, such as confidentiality, consent, and what and how to advise. Nevertheless, I prefer trained peer supporters from our community because the cultural differences with other communities make peer support more complicated. Hence, our organisation offers in-person peer support in our city. We also have a program called “round the table talk”, where we have conversations about mental health while having a coffee ceremony, and this makes it friendly, and people are open to talking about their experiences.*

(Sesen)

*We gather together weekly, have coffee, tea, and socialise and exchange ideas. Above all, we talk about mental well-being. Until now, the impact is that we are increasing in number, and peer influence is playing a role in supporting refugees’ and asylum seekers’ mental well-being. Above all, we have created a social network where they can support each other in different ways.*

(Basar)

The above excerpts demonstrate that MLCOs not only engage in capacity-building to overcome isolation but also blend scientific and traditional methods to support RASs in managing their mental health. Peer counsellors are trained to guide and advise, but the coffee ceremony also helps RASs share their experiences and listen to others. The coffee ceremony further suggests that MLCOs use ethnic cultural capital, such as cultural practices, to foster a conducive environment and support RASs’ mental well-being (Levanon, 2011). Additionally, both Sesen and Basar emphasise the importance of community organisation and development as core factors in guidance and counselling, suggesting that peer guidance should be integrated into this rather than being a separate effort. MLCOs’ initiative to organise and build a community can mobilize collective strength to improve community experiences.

*We support our clients through drop-in sessions, phone, or in person. We also have a coffee morning every month. We support them in collaborating with other organisations and signpost them based on their specific needs.*

(Hanna)

Hanna’s narrative indicates that MLCOs use online and face-to-face means to support RASs in need of guidance and counselling. However, this does not mean MLCOs can handle everything on their own. As Hanna’s story further shows, they work with other government and civic organisations, such as the NHS and Local Authorities, in knowledge exchange, and refer anything beyond their capacity to relevant agencies.

Many other participants, such as Awet, Giorgis, Habtom, Hanna, and Teages, supported Sesen’s and Basar’s arguments. In particular, Awet emphasised the importance of linguistic and cultural alignment, while Teages and Habtom highlighted the use of traditional approaches in guidance and counselling.

*Many Eritrean refugees and asylum seekers do not speak English and are new to UK culture. Hence, we need to address this to understand their stress and problems and help them become productive citizens.*

(Awet)

*We understand the Eritrean community as we are part of it. The cultural and linguistic advantage also helps us to contribute more effectively and make a real impact on their lives. We do better by combining traditional and modern guidance and counselling methods.*

(Habtom)

As can be seen, Awet’s and Habtom’s quotes suggest that Eritrean (peer) counsellors better understand the problems and, therefore, provide better solutions than an ordinary British counsellor who is not familiar with Eritrean culture. For instance, linguistic congruence allows RASs to express their feelings and build trust more easily. Additionally, most Eritrean RASs speak Tigrinya, share similar experiences of migration and trauma, and are mostly Christian or Muslim. This enables MLCOs to utilise their ethnic capital and develop more effective approaches to address migrants’ mental health issues, such as depression and anxiety.

The participants are right about one thing: RASs are not offered quality counselling and guidance services due to a lack of cultural competence (Pollard & Howard, 2021). In other words, culture is vital in guidance and counselling because it influences what is considered sensitive, acceptable, and deserving of support (Rigazio-DiGilio & Ivey, 2007). However, as in many Western countries, cultural and contextual factors are often overlooked when counselling services are provided to RASs in the UK (Pollard & Howard, 2021). This indicates that MLCOs have a key role in integrating cultural context and sensitivity into their work with RASs.

#### 5.2.4. Training and Skill Development

The findings show that Eritrean MLCOs offer various skill development programs to enhance the employment and employability prospects and experiences of RASs in the UK. These programs include training and workshops aimed at increasing refugees’ and asylum seekers’ knowledge and skills in areas such as information and communication technology (ICT), real estate investment, and healthy eating. For example, Finhas said:

*The first thing we did was to make CO3 known through its motivational speakers, then we started giving online educational sessions. CO3 identifies the limitations of refugees and asylum seekers and provides personal and professional development training and mentorship. It provides free and fee-based information and communication technology (ICT) and English language training, which runs for up to 6 months.*

(Finhas)

Many Eritreans lack ICT skills and English proficiency, which hampers their social and economic progress in the UK. Therefore, such training is essential for developing the skills of RASs, helping them compete in the country’s job market. This indicates that MLCOs mobilise migrants’ ethnic capital, especially human capital, to improve RASs’ experiences. Finhas further mentioned that most workshops and training sessions were run by volunteers and supported by mentorship programs. However, Finhas and Awet noted that a small fee and a certificate of completion were introduced for advanced training programs to boost participants’ interest and attendance. Although such an approach might provide a small incentive for volunteer teachers, it is important to recognise that it might also discourage some interested individuals from attending the training.

Sesen’s narrative below suggests that Eritrean RASs are trained to cook affordable, tasty, and healthy traditional dishes, as well as a variety of global dishes. For many RASs, finding traditional food is associated not only with physical and mental well-being but also with economic significance. Although many Eritrean and Ethiopian restaurants in the UK serve Eritrean dishes, they are primarily available in areas with large Eritrean and/or Ethiopian communities. The price is also unaffordable for many, especially asylum seekers. From Sesen’s perspective, that is why CO4 teaches how to prepare low-cost, healthy dishes in their own language: “*We educated refugees and asylum seekers to prepare healthy cultural food on a budget. This is beneficial, particularly for mothers and asylum seekers living on limited budgets*.”

As Sesen indicated, the training is essential for new mothers and asylum seekers, not only because of financial constraints, but also because they may lack prior experience and be far from family. Those who participate in the training also develop a social network that can help them support each other. Additionally, RASs can use their cooking skills to work in Eritrean and Ethiopian restaurants in the UK, where proficiency in the English language is not mandatory. In their explanations, both Finhas and Sesen underlined the essential role of ethnic capital in supporting RASs in their host countries.

Furthermore, many Eritrean MLCOs provide training and workshops in family formation and relationships, communication skills, social media use, and financial management (for example, see Mahtsen, 2025). These training courses and workshops are often used to develop RASs’ human capital, helping them navigate the complex, technology-driven modern world in their social and professional lives. Such training and workshops also echo what many participants, including Habtom, Teages, and Sesen, discussed about integrating RASs into the UK to access relevant services without being forced to abandon their culture of origin (Tsegay, 2020, 2022).

### 5.3. Challenges MLCOs Face

MLCOs face low community participation and limited resources, which affect their motivation and ability to perform tasks effectively and efficiently. The study participants indicated that various factors influence the participation of Eritrean RASs in activities organised by MLCOs.

*The challenges we have, particularly in our Eritrean community, are associated with people’s barriers like language, education, peer influence, and a lack of use of social media to follow our services. Many Eritreans also do not follow our activities or attend our services, such as courses and workshops. They do not read our advertisements, although they are written in Tigrinya and English.*

(Sina)

*We believe that every refugee must be equipped with English language and IT skills, and we organise good-quality classes. Although many register for the session, the attendance is low. The classes are free of charge. We do not have financial challenges, as our classes are online and have limited expenses.*

(Finhas)

The above excerpts suggest that refugees’ and asylum seekers’ capacity and interest are some of the factors that affect their participation in MLCOs’ activities. Sina noted that RASs’ interests are partly influenced by their educational qualifications, peer influence, and social media use. As shown above, the MLCOs are designed to address some of these challenges and equip RAS with English-language and IT skills. Overall, given that asylum seekers are not allowed to work (Mayblin & James, 2019; Tsegay, 2020), the MLCOs’ training and workshops are vital for making meaningful use of their time and for expanding their networks. However, as indicated above, many Eritrean RASs are dispersed across the UK and have insufficient digital skills and/or resources to access online services. This suggests that they might not be able to find the MLCOs’ event leaflets and/or attend the sessions.

*Although there are many users of our services, there are also times when participation in some events is low due to a lack of awareness of our organisation. We also do not have our own centre due to budget constraints; we rent various venues to host events.*

(Sesen)

Sesen’s statement highlights that many RASs are unaware of the services available and their benefits. That is why some MLCOs, as discussed earlier, are considering charging fees for certain services. Awet said, “We are now planning to charge for the classes they registered in order to increase the attendance and give the classes the value they deserve.” However, this approach would not address the low participation of RASs, as many may lack the financial means to pay for training. Instead, MLCOs should focus on promoting their organisations and services and on raising refugees’ and asylum seekers’ awareness.

Zemen and Keria argue that the political division of Eritreans into supporters and opposition of the Eritrean government has caused a rift in the community and affected the MLCOs’ activities. Although the MLCOs are non-political organisations, some of their members are politically active. This creates trust issues for some individuals who feel more comfortable socializing with and seeking support from Eritreans who share their political views. Sesen’s idea of “budget constraints” is supported by the majority of participants, including Giorgis, Hanna, and Zemen, who further indicated that MLCOs face a shortage of human and material resources.

*If we receive sufficient funding, we could organise more events and provide additional services, including special events for those who require support. However, funding is our main challenge.*

(Hanna)

*We offer support across various locations in the UK. Sometimes, it becomes too much to handle, with the jobs we have to do to survive in the UK, and we can only support as much as we can. The lack of time forces us to limit our support for the community primarily through online means. The organisation’s lack of staff makes it difficult for refugees to access the necessary support, and the remote delivery of support makes it challenging for people to explain their situation.*

(Zemen)

The participants mentioned that they lack sufficient funding to secure their own permanent space, hire staff to help coordinate activities, or cover the expenses of guest speakers’ travel and other costs. Although various grants are available for MLCOs in the UK, they are in high demand and highly competitive, considering the large number of charities in the country. As part of grassroots humanitarianism, MLCOs mostly depend on volunteers to run their activities. Nonetheless, many volunteers have full-time jobs and families to support. As Zemen explained, they often use their spare time to organise various activities and support RASs.

## 6. Discussion

The Eritrean MLCOs are small-scale organisations that emerged to address the challenges that RASs face in the UK. In most cases, in line with Dolska’s (2022) and Sandri’s (2018) arguments, the founders of MLCOs were often motivated to create these organisations to address gaps left by government agencies. This is evident in Sesen’s and Bereket’s stories, in which they established MLCOs after observing that government agencies were not providing relevant social and mental health services to RASs. Most MLCOs serve all Eritreans in the UK, though some focus on a specific religion, gender, or ethnic group. Participants like Habtom suggest that such organisations help facilitate restorative integration, which “seeks to restore human dignity jeopardised by the trauma involved in escaping danger and then seeking asylum” (Aldegheri et al., 2025, p. 39). In these cases, ethnic capital—defined as the collective human and social capital produced and used by members of an ethnic group (Kim, 2019)— is crucial for RASs to maintain their cultural roots while integrating into UK society and accessing vital services. It allows RASs to learn and receive support in their language from fellow Eritreans who understand their culture and experiences. However, the MLCOs’ focus on a specific religion or ethnic group might be perceived negatively, given the social and political divisions among diaspora Eritreans (Kidane, 2022; Hirt, 2015). As Zemen indicated, the Eritrean diaspora is already divided into supporters and opponents of the Eritrean government, an issue largely associated with the government’s one-party authoritarian nature (Shiker & Tsegay, 2025).

The research showed that MLCOs offer a variety of services to improve the socioeconomic, cultural, and mental well-being of RASs. They organise informational and workshop sessions and provide advice and advocacy to help RASs increase their knowledge and awareness of mental health, gender-based violence, and social media use, and to enhance their experiences in the UK. This is significant considering the challenges many refugees and asylum seekers face, for example, concerning gender-based violence (Tsegay & Tecleberhan, 2023) and digital literacy (Esenowo, 2022). Additionally, MLCOs offer various training and courses to improve refugees’ and asylum seekers’ skills in ICT, real estate development, and other fields. This suggests that MLCOs serve as learning and educational settings where RAS acquire various knowledge and skills, helping them become competitive in the UK job and business markets. The findings indicate that MLCOs’ services align with those provided by grassroots humanitarian or grassroots volunteer efforts (Lafaut & Coene, 2019). They rely on Eritrean refugees’, asylum seekers’, and other migrants’ ethnic capital, which serves as a form of human, cultural, and social capital (Shah et al., 2010). Furthermore, most MLCOs, often working with other partners, aim to create opportunities and influence policies and practices that impact RASs. This shows that MLCOs participate in a variety of activities, balancing humanitarian efforts with activism (Muehlebach, 2019; McGee & Pelham, 2018; Vandevoordt, 2019), and they act as vehicles for social and political change (Lafaut & Coene, 2019).

Some of the services provided by MLCOs are essential, especially for new asylum seekers who are not allowed to work (Gower et al., 2024) and are dispersed to remote areas across the UK (Darling, 2022; Tsegay, 2020). Therefore, the emotional and material support they receive helps them feel cared for and reduces their stress and anxiety, which they face during their migration journey and the lengthy, complex asylum process (Tsegay, 2020, 2022). Whenever possible, MLCOs try to blend scientific and traditional methods of guidance and support to RASs and address their needs. One significant example mentioned by many participants is the use of a traditional ‘coffee ceremony’ to help RASs. This is a traditional means of talk therapy in which many share their thoughts, feelings, and experiences and are listened to without judgment. Peer counselling also helps RASs receive culturally sensitive support in their language from fellow Eritreans who understand their experiences. This approach addresses the lack of culturally relevant guidance and counselling by mainstream British institutions due to a lack of cultural competence (Pollard & Howard, 2021).

However, Eritrean MLCOs often lack sufficient human and material resources, and most activities are run by volunteers, as is common in much grassroots humanitarian work (Muehlebach, 2019; Sandri, 2018). In addition, the participants noted that many Eritreans in the UK are unaware of these services, especially given the UK’s dispersal policy (Darling, 2022; Tsegay, 2020). Low community participation and awareness are also linked to refugees’ lack of time and asylum seekers’ limited digital technology skills or access. Without mobile internet access, Eritrea has one of the lowest digital penetration rates globally (Tsegay, 2016, 2020). The findings suggest that many RASs fail to complete the training they begin for various reasons, such as a lack of time or awareness. As a result, some MLCOs are considering charging for their training, which could further reduce participation among RASs, as they may be unable to afford the fees. Asylum seekers are not permitted to work (Gower et al., 2024) but instead receive a limited allowance to cover essential living expenses (Gower, 2021).

## 7. Conclusions

In this article, we have underscored the extreme vulnerability of RASs and articulated the MLCOs’ robust advocacy and support systems to protect their rights and address their needs. Migrants, especially RASs, use their experiences of vulnerability to support and improve the socioeconomic and cultural conditions of those who arrive after them. This is the main reason many migrants have established MLCOs or volunteer with the organisations. Drawing on “grassroots humanitarianism” (Dolska, 2022; Sandri, 2018), the findings reiterated that MLCOs are essential for filling gaps that government agencies cannot. Although the MLCOs are run by volunteers and have limited funding, they deploy the ethnic capital to provide culturally sensitive services to RASs in their own language (Borjas, 1992; Kim, 2019). They use RASs’ and other migrants’ social, cultural and human capital to provide activities associated with community awareness and inclusion, advocacy, guidance and counselling, and skill development. In doing so, MLCOs serve as sites for learning and education.

Contrary to the perception that migrants are passive and a burden on state institutions (Tsegay, 2026), this article shows that migrants organise and work to address their challenges at the community level. Overall, MLCOs play a significant role in improving the lives of RASs in the UK and, as Sesen explained, their contribution is even greater during crises such as the UK–Rwanda partnership to send asylum seekers to Rwanda (see Parker & Cornell, 2024). However, RASs live in a state of uncertainty and vulnerability, often exacerbated by continuous and harsh immigration changes. The recent UK immigration change to extend the time for indefinite leave to remain and restrict family reunion and universal credit for RASs is an evident example of hostilities aimed at ‘making it harder to move to and settle’ in the country (see Gower & McKinney, 2025). This suggests that MLCOs will be more vital than ever with the growing anti-immigrant protests and laws, which could exacerbate refugees’ and asylum seekers’ destitution, fear, and anxiety.

This article provides a better understanding of how MLCOs contribute to improving refugees’ and asylum seekers’ experiences in their host countries and to their becoming productive members. It also helps shape MLCOs’ practices to acknowledge and enhance RASs’ human capital and ultimately improve their access to relevant services. However, the article has some limitations. It is based on small focus group discussions with leaders of Eritrean MLCOs. A larger study that includes the voices of RASs and other communities is needed to ensure comparative analysis and advance understanding of these issues.

## Figures and Tables

**Table 1:** Focus group participants’ information.

Participant (Pseudonym)	Gender	Organisation (Pseudonym)
Awet	Female	CO3
Basar	Female	CO2
Bereket	Male	CO3
Elen	Female	CO3
Finhas	Male	CO3
Giorgis	Male	CO2
Habtom	Male	CO6
Hanna	Female	CO9
Keria	Female	CO9
Saron	Female	CO7
Sesen	Female	CO4
Sina	Female	CO1
Teages	Male	CO5
Zemen	Male	CO8

## Data Availability

The original contributions presented in this study are included in the article. Further inquiries can be directed to the corresponding author.
